# Biocorrosion and Cytotoxicity Studies on Biodegradable Mg-Based Multicomponent Alloys

**DOI:** 10.3390/bioengineering11060621

**Published:** 2024-06-18

**Authors:** Priya Sudha, Khin Sandar Tun, Jisha Pillai, Mainak Dutta, Manoj Gupta, Vincent Shantha Kumar

**Affiliations:** 1 Department of Mechanical Engineering, Birla Institute of Technology and Science Pilani (BITS Pilani), Dubai Campus, Dubai 345055, United Arab Emirates; 2 Department of Mechanical Engineering, National University of Singapore, Singapore 117576, Singaporempegm@nus.edu.sg (M.G.); 3 Department of Biotechnology, Birla Institute of Technology and Science Pilani (BITS Pilani), Dubai Campus, Dubai 345055, United Arab Emirates

**Keywords:** magnesium-based multicomponent alloys, disintegrated melt deposition (DMD) technique, biocorrosion studies, cytotoxicity, antitumor cells, scanning electron microscopy (SEM), energy-dispersive X-ray spectroscopy (EDS)

## Abstract

Magnesium-based multicomponent alloys with different compositions, namely Mg_60_Al_20_Zn_5_Cu_10_Mn_5_ (Mg60 alloy), Mg_70_Al_15_Zn_5_Cu_5_Mn_5_ (Mg70 alloy), and Mg_80_Al_5_Cu_5_Mn_5_Zn_5_ (Mg 80) alloys, were prepared using the disintegrated melt deposition technique. The DMD technique is a distinctive method that merges the benefits from gravity die casting and spray forming. This approach facilitates high solidification rates, process yields, and reduced metal wastage, resulting in materials with a fine microstructure and minimal porosity. Their potential as biodegradable materials was assessed through corrosion in different simulated body fluids (SBFs), microstructure, and cytotoxicity tests. It was observed that the Mg60 alloy exhibited low corrosion rates (~× 10^−5^ mm/year) in all SBF solutions, with a minor amount of corrosive products, and cracks were observed. This can be attributed to the formation of the Mg_32_(AlZn)_49_ phase and to its stability due to Mg(OH)_2_ film, leading to excellent corrosion resistance when compared to the Mg70 and M80 alloys. Conversely, the Mg80 alloy exhibited high corrosion rates, along with more surface degradation and cracks, due to active intermetallic phases, such as Al_6_Mn, Al_2_CuMg, and Al_2_Cu phases. The order of corrosion resistance for the Mg alloy was found to be ASS > HBSS > ABP > PBS. Further, in vitro cytotoxicity studies were carried out using MDA-MB-231 tumor cells. By comparing all three alloys, in terms of proliferation and vitality, the Mg80 alloy emerged as a promising material for implants, with potential antitumor activity.

## 1. Introduction

Over the past few decades, metallic materials such as stainless steel, Co–Cr, and titanium alloys have been commonly used in orthopedic applications, due to their excellent corrosion resistance and mechanical properties compared to ceramic and polymer materials [1]. Even though they meet the requirements of biomaterials, they remain in the body after the wound healing process, leading to long-term endothelial dysfunction [2]. When these materials are used in long-term applications, these materials emit corrosive ions that have a detrimental effect on the body and exhibit undesirable mechanical properties [3]. Moreover, metallic implants must be removed from the body through secondary surgery, once the tissue has healed. Therefore, it is important to develop next-generation metallic implants that degrade in the body without causing any adverse effects to the human body. Recently, biodegradable metallic materials, such as iron, zinc, and their alloys, as well as Mg and its alloys, have been developed [4,5]. Among these, magnesium alloys are promising materials that have recently received more and more significant interest as potential biodegradable materials for biomedical applications [6].

Magnesium is one of the most attractive biodegradable elements with excellent favorable properties, such as a low density (1.74 g/cm^3^), a high strength/weight ratio, effective heat dissipation, good damping, good electromagnetic shielding capabilities, biocompatibility, and it is essential for human metabolism [7]. This interest arises from the mechanical properties of Mg alloys, which have a resemblance to those of natural bone, and are less dense compared to other metallic biomaterials [1,8]. This characteristic minimizes the stress shielding effect and the consequent osteopenia. In addition to its mechanical properties, the biodegradable nature of Mg alloys has caused lots of interest among researchers. The use of these biodegradable implant materials reduces the necessity for secondary surgery to remove the implant after the tissue has healed, resulting in lower medical costs [9]. This helps to reduce the problems that are caused by permanent implants, such as endothelial dysfunction, persistent physical irritation, and local chronic inflammatory reactions [10]. Furthermore, magnesium plays a crucial role in stabilizing biological processes in the human body, plants, and animals. The recommended daily intake of Mg for adults is 240–420 mg/day, which is up to 52.5 times higher than the recommended intake for iron (8–18 mg/day) and zinc (8–11 mg/day).

The primary concern about the use of Mg alloys as an implant is its rapid degradation during healing, leading to a reduction in its mechanical strength during the healing time [11,12]. A sufficient corrosion rate is necessary for implants to biodegrade in bodily fluid. The application of Mg implants is limited due to the significant degradation rate of pure Mg at the physiological pH level (7.4–7.6) and in the high chloride environment of physiological systems [13]. This leads to a harmful interaction with biological organisms, and also a loss of mechanical integrity when in contact with high pH levels and high chlorine environments of simulated body fluids [8]. In aqueous environments, the corrosion of Mg alloys is attributed to a higher electrode potential of −2.3 V. This elevated corrosion rate results in the loss of its mechanical properties, leading to premature implant failure and hindering the intended tissue healing process [14]. The second concern is the evolution of a high rate of hydrogen due to corrosion [15], thus, leading to a blockage of blood pathways and a negative effect on neighboring tissues [16]. Because of these serious problems, the usage of a Mg-based implant for biomedical application is limited to 6–8 weeks. On the other hand, vital recovery processes typically take about 12 weeks [17]. Despite these challenges, magnesium implants have demonstrated their effectiveness in promoting the development of new bone, when used as bone fixings.

Therefore, enhancing the corrosion resistance of Mg alloys for bioimplant applications is imperative. Three methods can be employed to achieve the necessary corrosion resistance and mechanical properties of Mg. The first method involves the addition of alloying elements, which play a crucial role in enhancing structural properties, corrosion resistance, and biocompatibility [12]. However, for implant applications, the careful selections of alloying elements in regard to Mg is essential to prevent any adverse effects on the human body [18]. The second method entails the development of novel composite material with the Mg alloy [11]. The third method focuses on surface modification and fabrication techniques, which can effectively improve corrosion resistance and tribological properties.

Therefore, the motivation for the current study is to enhance the corrosion resistance of Mg alloys for bioimplant application, which is achieved through alloying elements and surface modification techniques. In this research, a novel Mg-based multicomponent system has been developed using alloying elements, such as Al, Zn, Cu, and Mn, along with an advanced fabrication technique. The addition of an alloying element can modify the nature of the passive film, by influencing the current density of both anodic and cathodic reactions. Additionally, these alloying elements contribute to the formation of new intermetallic/disordered structures. This disordered structure helps increase the overall entropy of the alloy, resulting in the formation of both hard and soft phases. These phases exhibit high strength and ductility [19], which in turn affects the corrosion mechanism.

Aluminum(Al) stands out as the most commonly used alloying element in regard to magnesium. Mg–Al based alloys find application in the development of biodegradable implants owing to their excellent castability, acceptable corrosion resistance, and mechanical properties [20]. The enhanced corrosion resistance is attributed to the formation of the Mg_17_Al_12_ intermetallic compound, which contributes to stabilizing the alloy microstructure [21]. Another important consideration to improve the corrosion resistance in Mg–Al alloys is the need to maintain impurities (Fe, Ni, and Cu) within a certain tolerance limit [22]. Exceeding these limits can lead to the formation of localized corrosion, which is detrimental to the mechanical integrity of the implants. However, it is important to note that aluminum is a neurotoxic element. If a person’s daily intake of Al surpasses the maximum allowable limits, it can lead to Alzheimer’s disease or dementia in patients [23]. Based on previous reports, Al content exceeding 5 wt.%, as seen in alloys like AZ91 and AM61 alloys, can have adverse effects on neurons and osteoblast cells [24]. The maximum permissible aluminum (Al) content in magnesium (Mg) alloys for implant applications varies depending on the specific alloy composition, regulatory guidelines, and biocompatibility requirements. This limitation is maintained within narrow ranges to avoid the risk of neurotoxicity and ensure optimal biocompatibility when an alloy is implanted in the human body. Therefore, manufacturers and researchers need to adhere to these limits to ensure the safety and efficacy of Mg alloy implants for medical use. Interestingly, observations indicate that AZ31, AZ91, and AZ61 alloys displayed an improvement in their corrosion potential values after immersion for 16 and 24 days in SBF, PBS, and NaCl solutions compared to pure Mg [20].

Zinc and copper play essential roles in increasing corrosion resistance, especially when utilized alongside magnesium in biomedical applications. Zinc is a crucial trace mineral for the human body, naturally present in cells throughout the body. Nutritionally essential, an appropriate amount of zinc improves the corrosion resistance of magnesium alloys by forming a protective layer on the surface, which restricts corrosion [25]. This protective layer acts as a barrier against corrosion, which improves the lifespan of the implant. Additionally, zinc can act as a sacrificial anode, preferentially corroding instead of the magnesium matrix, thereby protecting the alloy from corrosion [26,27]. This makes zinc the second most commonly used alloying element in magnesium after aluminum. Aidin et al. [28] discuss and study the improved corrosion resistance of biodegradable magnesium (AZ91) in simulated inflammatory conditions, through the application of a betamethasone sodium phosphate (BSP) layer on plasma electrolytic oxidation (PEO) coatings for orthopedic implant application. The electrochemical test results revealed that a significant improvement in the corrosion resistance was observed in the PEO/BSP-coated Mg alloy sample in simulated inflammatory conditions, compared to the uncoated Mg alloy. This is mainly due to the fact that the BSP layer seals the PEO coating, enhancing its barrier performance in acidic environments.

Copper is another cost-effective material and an important element in bone resorption and the immune system due to its antibacterial nature, which destroys bacterial cells. Generally, in stainless steel and cobalt-based alloys, copper has been used as an alloying element to increase bacterial resistance [29]. However, the usage of Cu in Mg alloys has not been applied more widely, due to the fact that Cu increases the current density in the cathode region [30]. Recent studies have found that adding a small amount of copper to magnesium alloys can enhance its degradation characteristics, resulting in improved antibacterial properties, enhanced biocompatibility, and better mechanical performance [26,27]. Consequently, copper emerges as a promising candidate for various biological applications. Moreover, copper contributes to corrosion resistance by enhancing the passivation of the magnesium alloy surface. Copper ions promote the formation of a stable oxide layer on the surface, acting as a barrier against corrosion. Additionally, copper’s antimicrobial properties can help prevent infections around the implant site [31].

When zinc and copper are combined with magnesium, a synergistic effect can occur that further enhances the corrosion resistance. The addition of Zn and Cu leads to the formation of a complex microstructure, enhancing the uniformity and stability of the protective oxide layer. This leads to an enhancement of the alloys’ resistance to corrosion in physiological environments [32].

Similarly, the addition of Mn to Mg, as a binary alloying element, exhibited no significant change in the corrosion rate. It is always essential to add Mn to Mg–Al and Mg–Al–Zn systems [33]. Manganese is one of the most important alloying elements in the human body, contributing to maintaining healthy bone structure, preventing osteoporosis, and regulating bone metabolism [34]. To diminish the influence of iron in Mg alloys, the Fe/Mn ratio should be limited to 0.032 (maximum limit). Beyond this limit, corrosion rates will increase significantly [35].

When combining alloying elements, such as Al, Zn, Cu, and Mn, synergistic effects [36] can result when their individual properties complement each other, leading to improvements in the overall performance of the alloy. For example, the addition of Cu and Mn to Al–Zn can enhance its strength, corrosion resistance, and workability [37,38,39]. However, antagonistic effects [36] may occur if certain elements are combined in a way that result in the formation of undesirable intermetallic compounds, leading to the weakening of the alloy’s mechanical properties and corrosion resistance. Excessive amounts of certain elements can also lead to increased brittleness or processing difficulties.

Finally, it is concluded that each alloying element provides unique advantages to bioimplant applications. Careful consideration of the composition and interaction between alloying elements is important to harness the synergistic effects, while avoiding antagonistic effects. The exact composition of the alloy is essential to optimize the mechanical properties, corrosion resistance, biocompatibility, and long-term performance in bioimplant and biomedical applications.

Thus, the current research aims to assess the suitability of novel Mg–MCA alloys, such as Mg_60_Al_20_Zn_5_Cu_10_Mn_5_ (Mg60 alloy), Mg_70_Al_15_Zn_5_Cu_5_Mn_5_ (Mg70 alloy), and Mg_80_Al_5_Cu_5_Mn_5_Zn_5_ (Mg 80 alloys), for various biomedical applications. To achieve this, the alloys were subjected to potentiodynamic polarization tests and electrochemical impedance spectroscopy in different simulated body fluid solutions. Additionally, in vitro studies were conducted to evaluate their biocompatibility properties.

## 2. Experimental Details

### 2.1. Fabrication and Sample Preparation

Cast ingots of the novel Mg-based multicomponent alloys (Mg–MCA alloy), such as Mg_60_Al_20_Zn_5_Cu_10_Mn_5_ (Mg60 alloy), Mg_70_Al_15_Zn_5_Cu_5_Mn_5_ (Mg70 alloy), and Mg_80_Al_5_Zn_5_Cu_5_Mn_5_ (Mg80 alloy) (at. %) alloys, were fabricated using the disintegrated melt deposition (DMD) technique. This process involved utilizing a resistance heating furnace in the presence of argon gas to prevent oxidation of the alloys [40].

After successful fabrication of the novel Mg-based multicomponent alloys (Mg60, g70, and Mg80 alloys), they were cut into cuboid shapes (1 × 1 × 1 cm) using a diamond cutting machine. Subsequently, they were polished with silicon carbide (SiC) grinding paper, ranging from 200 to 1200 grit size. After each grinding step, the samples were rotated by 90° to remove scratches and unwanted layers. Next, the specimens were cleaned in acetone, washed with distilled water, and dried, before performing electrochemical measurements and microstructural analysis.

### 2.2. Electrochemical Characterization

To examine the corrosion behavior of the Mg–MCA alloys, potentiodynamic polarization tests were conducted with a Metrohm Autolab 204 Potentiostat. The tests were performed using three electrode cells, where the platinum wire served as the counter electrode (CE), the saturated calomel electrode (SCE, USCE = 241 mV) served as the reference electrode (RE), and the test sample as the working electrode (WE). The testing spanned from the OCP to both the anodic and cathodic regions, at a scan rate of 0.000167 v/s. These tests were carried out at room temperature in four different simulated body fluid solutions (SBFs): artificial blood plasma solution (ABP, pH 7.4), phosphate-buffered saline solution (PBS, pH 7.4), artificial saliva solution (ASS, pH 6.4), and Hanks’ balanced saline solution (HBSS, pH 7.4). All the electrolyte solutions were prepared using analytical grade reagent chemicals and distilled water. The chemical composition of the four simulated body fluids is outlined in Table 1. Electrochemical impedance spectroscopy (EIS) was performed using VersaSTAT 3 Potentiostat, in the same three-electrode cell configuration, to generate Nyquist and Bode plots for each electrolyte solution. The AC frequency ranged from 10 kHz to 1 Hz, providing sufficient coverage for impedance property testing, with a 10 mV peak-to-peak sine wave serving as the excitation signal. The impedance spectra were analyzed using EC-Lab software [41]. Subsequently, a suitable equivalent circuit was selected to fit the best curve. Detailed experimental procedures are elaborated elsewhere [42].

### 2.3. SEM–EDS Analysis

Following a successful investigation into the corrosion behavior of the Mg–LEA alloy, the corroded surface was analyzed using scanning electron microscopy (SEM) (model: JEOL JSM-6010) to gain insights into the surface morphologies. Additionally, elemental mapping of the corroded surface was performed using energy-dispersive X-ray spectroscopy (EDS), which was integrated with the SEM for a comprehensive characterization of the elemental distribution.

### 2.4. In Vitro Studies: Cytotoxicity Assay Using MTT (3-(4,5-Dimethylthiazol-2-yl)-2,5-Diphenyltetrazolium Bromide)

Human breast tumor cell lines (MDA-MB-231) were used in an in vitro cell culture experiment. The cells were cultured in Dulbecco’s modified eagle medium (DMEM), supplemented with 10% fetal bovine serum (FBS), at 37 °C in a humidified atmosphere with 5% CO_2_.

The Mg alloy samples were sterilized in 70% ethanol for 1 h and were subsequently rinsed with 0.85% NaCl solution. Following sterilization, these samples were incubated in the DMEM medium, with a surface area-to-extraction medium ratio of 1 cm^2^/mL, in a humidified atmosphere with 5% CO_2_ at 37 °C for 72 h. After incubation, the supernatant fluid was extracted and centrifuged at 3000 rpm for 5 min to prepare the samples for the cytotoxicity tests. In the experimental design, the control group utilized the DMEM medium as the negative control and the 10% dimethyl sulfoxide (DMSO) medium as the positive control. The cells were incubated in 96-well plates, at a cell density of 2 × 10^4^ cells/mL in each well, and were allowed to incubate for 24 h to facilitate attachment with the cells. Subsequently, the medium was replaced with 100 μL of alloy extraction (Mg60, Mg70, and Mg80) medium. The cells were then incubated in a humidified atmosphere with 5% CO_2_ at 37 °C for 24, 48, and 72 h, respectively. Observations of the 96-well cultured plates were made using an Olympus inverted microscope. Following this, the extraction medium was replaced with 200 μL of cultured medium, containing 20 μL of MTT solution (5 mg/mL PBS). The samples were incubated with MTT for 4 h at 37 °C, and the formazan reaction products were dissolved in 150 μL dimethyl sulfoxide (DMSO) for 20 min in a humidified atmosphere. The spectrophotometrical absorbance of the samples was measured using a microplate reader (Bio-RAD680) at 570 nm, with a reference wavelength of 630 nm. Each experiment was conducted in triplicate, along with the control, and the cell viability (%) was calculated using formula [43]:

$$Viability\;\%=\frac{100-{OD}_{570e}}{{OD}_{570b}}$$

where OD_e_ is the absorption of the sample and OD_b_ is the absorption of the vehicle control.

## 3. Results and Discussion

### 3.1. Potentiodynamic Polarization Measurements and Surface Morphologies

**Figure 1:**
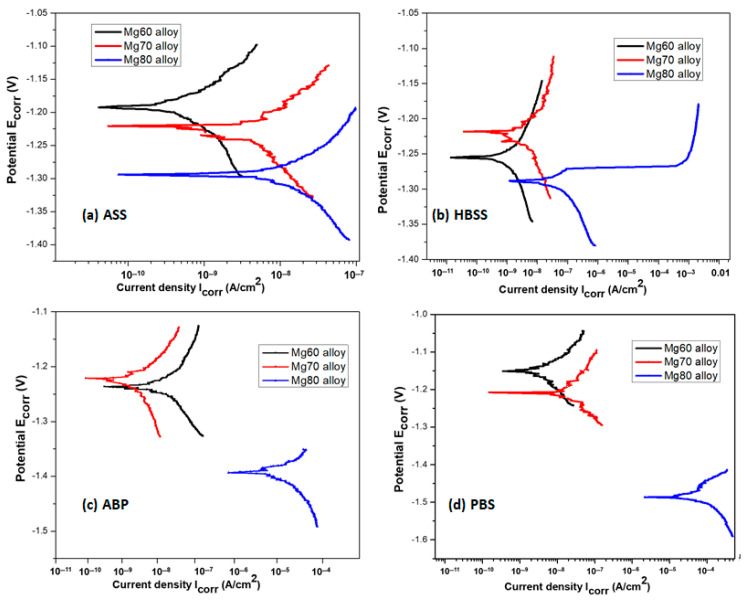
Polarization curves of Mg60, Mg70, and Mg80 alloys in various SBF solutions.

The potentiodynamic polarization curves of the Mg–MCA alloys (Mg60 alloy, Mg70 alloy, and Mg80 alloy) tested in various SBF solutions are illustrated in Figure 1a–d and the corresponding corrosion parameters are summarized in Table 2. The results, as depicted in Figure 1a–d, indicate a notable passivation tendency in the anodic region for almost all the polarization curves in various SBF solutions. From the results (Figure 1a,b,d, and Table 2), except for ABP (Figure 1c and Table 2), the polarization curves of the Mg60 alloy displayed low current densities in PBS, ASS, and HBSS solutions, indicating good corrosion resistance. As the Mg content increases from Mg60 to Mg70 in PBS, ASS, and HBSS solutions, the corrosion potential (E_corr_) becomes more negative with a concurrent increase in the corrosion current (I_corr_), as observed in Figure 1a,b,d, and Table 2. This indicates that the alloy is under cathodic control, indicating high degradation rates. Conversely, in the case of the ABP solution (Figure 1c and Table 2), the current density (I_corr_) value of the Mg60 alloy is slightly higher than that of the Mg70 alloy. This implies that Mg70 exhibited favorable passivation behavior in the ABP solution.

With a further increase in the Mg content from Mg70 to Mg80 in all the four SBF solutions, a significant increase in I_corr_ can be observed and E_corr_ moved towards the more negative side (Figure 1a–d and Table 2). This notable increment in the I_corr_ value for the Mg80 alloy suggests a high rate of metal dissolution at E_corr_. This high metal dissolution causes rapid degradation of the alloy. Among all the solutions, i.e., ASS, PBS, ABP, and HBSS solutions (Figure 1a–d), all the three Mg–MCA alloys displayed better passivity and excellent corrosion resistance in the ASS solution and high corrosion rates were observed in the PBS solution. This high degradation can be attributed to the elevated concentration of Cl^−^ ions (Cl^−^) in the ABP, PBS, and HBSS solutions compared to ASS (Table 2). Previous studies have indicated that sulphate (SO_4_^2−^) and phosphate (PO_4_^3−^) ions can increase the metal dissolution of Mg alloys [44], with chloride ions (Cl^−^) being identified as more aggressive and detrimental to the alloy. The primary factors influencing the corrosion behavior of Mg alloys in an aqueous environment are the chemical composition of the SBF solution and its pH level [45]. As seen in Table 1, the chemical composition of the ABP, PBS, and HBSS solutions at pH 7.4 can significantly accelerate magnesium dissolution compared to ASS at pH 6.2. This is primarily due to the lower Cl^−^ ion concentration in the ASS solution (NaCl-1.5 g/L) and the absence of sulphate (SO_4_^2−^) and phosphate (PO_4_^3−^) ions. Based on the previous analysis, Mg–MCA alloys exhibit superior corrosion resistance in alkaline solutions (pH < 7).

**Figure 2:**
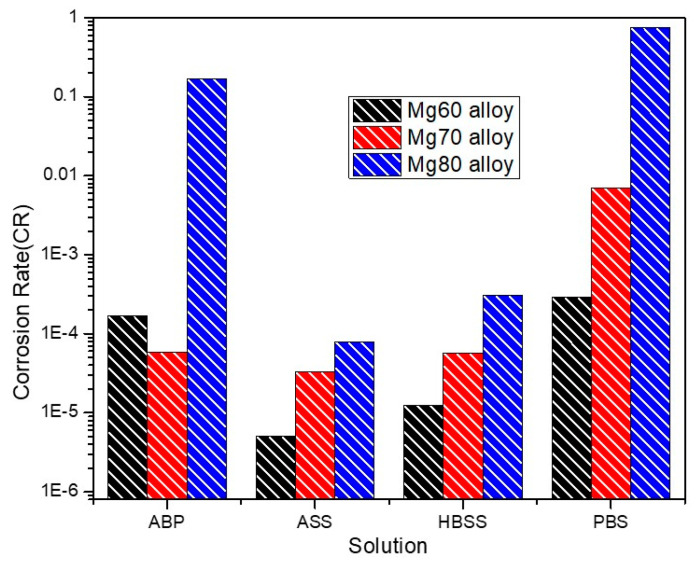
Corrosion rate (mm/year) of Mg60, Mg70, and Mg80 alloys in SBF solutions.

Furthermore, the corrosion rates (C.R) in mm/year of the Mg60, Mg70, and Mg80 alloys in SBF solutions are calculated using I_corr_, with the help of Faraday’s Law [46], and the results are depicted in Figure 2.

$$Corrosion\;Rate\;(C.R)=3.27\times 10^{-3}*I_{corr}(µA/{cm}^{2})\times \left(\frac{E_{w}}{\rho }\right)\;mm/year$$

where 3.27 × 10^−3^ is Faraday’s constant, I_corr_ is the corrosion current density (µA/cm^2^), E_w_ is the equivalent weight of the corroding material, and ρ is the density of the material in g/cm^3^.

Compared to all three Mg–MCA alloys, the Mg60 alloy displays the least corrosion rate values, signifying excellent corrosion resistance in all SBF solutions, except the ABP solution. Previous reports suggest the maximum degradation rate for implants in SBF solutions is 0.5 mm/year [47]. In comparison to previously reported alloys, the current experimental results for the Mg60 and Mg70 alloys display very low corrosion rates (~× 10^−5^ mm/year) in all SBF solutions (Figure 2). Furthermore, in comparison to ASS at pH 6.4, the Mg alloy’s corrosion rate was found to be high in the HBSS solution, followed by the ABP and PBS solutions at pH 7.4 (Figure 2). Whereas in the case of the Mg80 alloy in the PBS solution, the corrosion rate was notably high at 0.74 mm/year (Figure 2). Among all the results, the corrosion was more noticeable in the PBS solution. The corrosion rates of the Mg alloys can be ranked in the following order: ASS <HBSS < ABP < PBS.

The observed increase in the corrosion behavior of the Mg–MCA alloys (Mg60, Mg70, and Mg80) across the four different SBF solutions with varying pH levels, particularly in regard to the PBS solution, is attributed to the presence of chlorine ions. These ions facilitate the formation of an MgCl_2_ layer, which can disrupt the passive layer of magnesium hydroxide (Mg(OH)_2_), leading to the breakdown of the passive layer and the increased dissolution of Mg alloys. This disruption promotes localized corrosion mechanisms, such as pitting and crevice corrosion. Additionally, the composition of alloying elements can influence the corrosion dynamics in different pH environments. In the Mg60, Mg70, and Mg80 alloys, higher corrosion rates are observed with increasing Mg content from the Mg60 to the Mg80 alloy. The higher concentration of Mg content in the Mg80 alloy makes it more challenging to form a passive layer (Mg(OH)_2_), due to the increased reactivity of Mg compared to the Mg60 and Mg70 alloys. This leads to less protection of the metal from corrosion. Furthermore, the higher Mg content might cause less stable intermetallic phases or alloy microstructures to form, which can promote corrosion initiation and propagation.

**Figure 3:**
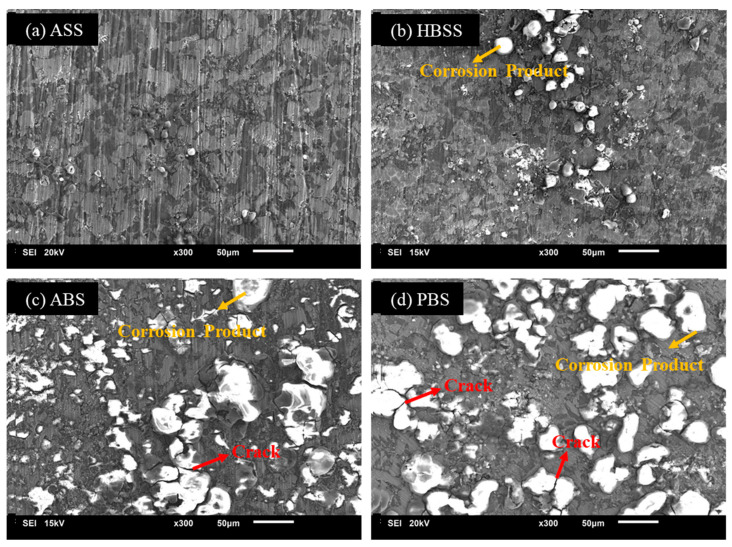
SEM micrographs (**a**–**d**) showing morphologies of corroded surface of the novel Mg60 alloy in SBF solutions.

The surface morphologies of the Mg60 alloy were observed using SEM, and the results are displayed in Figure 3. The SEM images (Figure 3a–d) provide insights about the degradation behavior of the Mg60 alloy in each SBF solution. Figure 3a shows the sample immersed in the ASS solution at pH 6.2, with no surface defects observed on the sample surface. This suggests that the alloy was completely protected by the passive layer, indicating excellent corrosion resistance. Further, in the HBSS solution (Figure 3b), a small amount of surface degradation was found on the sample surface, implying a good level of corrosion resistance. Figure 3c represents the surface morphologies of the Mg60 alloy sample tested in the ABP solution. Deeper cracks with large traces of white irregular corrosive products were clearly noticed on the alloy surface. Previous studies have shown that a Mg(OH)_2_ protective layer forms easily on Mg alloys when exposed to a humid environment [48]. However, in the case of the Mg60 alloy in the ABP solution, the protective layer is continuously breaking down due to the presence of a high concentration of Cl^−^ ions, along with other aggressive elements like PO_4_^3−^ and SO_4_^2−^ ions, even though the pH level is 7.4 (from Table 1). The presence of aggressive ions, such as Cl^−^, along with PO_4_^3−^ and SO_4_^2−^ ions, rapidly increases the degradation rate. Thus, the Mg–LEA alloy in ABP displays a high corrosion rate compared to the HBSS solution. In the PBS solution (Figure 3d), similar to ABP, more surface damage and deeper cracks were detected on the alloy surface. The SEM images of the Mg60 alloy, displayed in Figure 3, correlate well with polarization results, showing that the ASS solution at pH 6.2 exhibits good corrosion resistance compared to other solutions at pH 7.4 (ABP, PBS, and HBSS).

**Figure 4:**
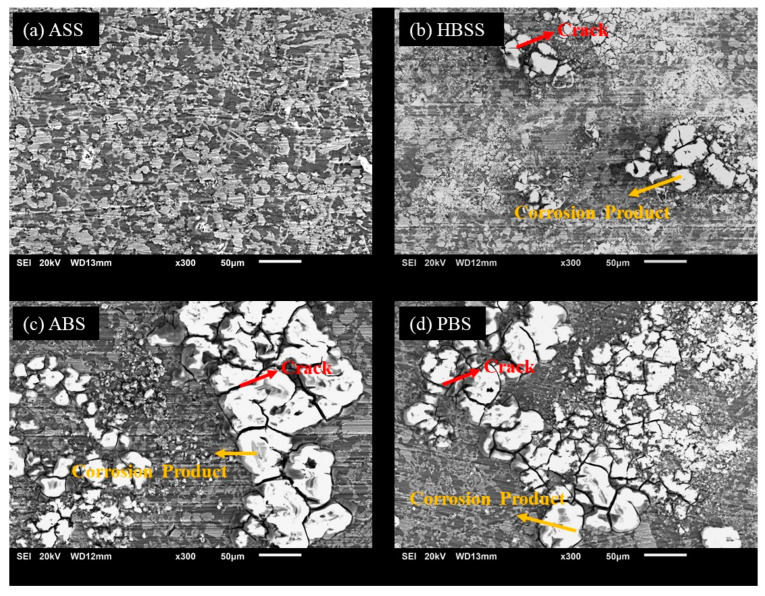
SEM micrographs (**a**–**d**) showing morphologies of corroded surface of the novel Mg70 alloy in SBF solutions.

The Mg70 alloy samples were analyzed using SEM to examine their surface morphologies in different body fluid conditions, as shown in Figure 4. The results demonstrated that no surface defects were observed on the alloy surface when immersed in the ASS solution at pH 6.4 (Figure 4a). However, in the HBSS solution at pH 7.4 (Figure 4b), minor surface degradation, with fine cracks, was observed on the sample surface. In the case of the ABP and PBS solution (Figure 4c,d), the Mg(OH)_2_ surface layer was broken down, causing white irregular corrosive products with deeper cracks that were clearly observed. It can be noted that the white corrosion product of the Mg70 alloy becomes denser compared to the Mg60 alloy (Figure 3). The increase in Mg content with low alloying elements and the chemical composition of the SBF solutions contribute to the high corrosion rates in the Mg70 alloy.

**Figure 5:**
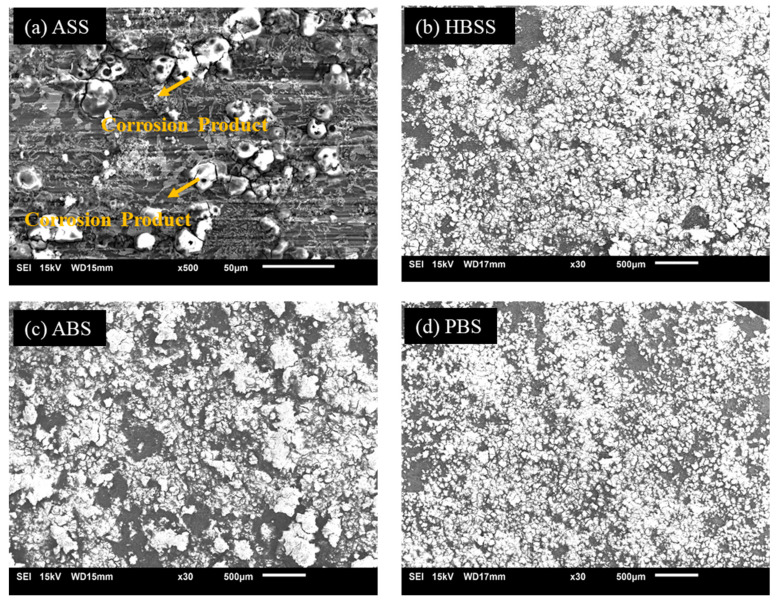
SEM micrographs (**a**–**d**) showing morphologies of corroded surface of the novel Mg80 alloy in SBF solutions.

Figure 5 shows the SEM micrographs of the Mg80 alloy tested in different SBF solutions. The results reveal that the Mg80 alloy in the ASS solution (Figure 5a) displays minor corrosive defects on the sample surface. However, in the HBSS (Figure 5b), ABP (Figure 5c), and PBS solutions (Figure 5d), the alloy surface was severely corroded, showing a large trace of white corrosion product and developed cracks, indicating poor corrosion resistance of the Mg80 alloy. This was mainly due to the increase in the Mg content and the chemical composition of the SBF solutions, leading to the overflow of evolved hydrogen bubbles. Simultaneously, Cl^−^ ions quickly moved onto the alloy surface, breaking the Mg(OH)_2_ passive layer. As a result, the surface film layer was completely dissolved, and also larger, deeper cracks were observed, indicating high corrosion rates. In comparison to the Mg60 alloy (Figure 3) and the Mg70 alloy (Figure 4), very high metal dissolution was observed in the Mg80 alloy (Figure 5) in all four different body fluid solutions. The SEM morphologies of the Mg60, Mg70, and Mg80 alloys (Figure 3, Figure 4 and Figure 5) correlate well with the polarization results (Figure 1 and Figure 2).

The present results on the novel magnesium-based multicomponent alloys (Mg–MCA), namely the Mg60 alloy, Mg70 alloy, and Mg80 alloy, were compared with previously reported results for phosphate-buffered and Hank’s solutions [49,50], as shown in Table 3. The current results for the three Mg–MCAs in PBS media displayed I_corr_ (A/cm^2^) values of 1.97 × 10^−8^ A/cm^2^ (Mg60), 4.30 × 10^−9^ A/cm^2^ (Mg70), and 4.40 × 10^−5^ A/cm^2^ (Mg80) (Table 2). In comparison, a Mg–Mn–Zn alloy had an I_corr_ of 7.917 × 10^−5^ A/cm^2^, as reported in Table 3 [49]. This indicates that the corrosion resistance of the Mg60 and Mg70 alloys were significantly superior to that of the Mg–Mn–Zn alloy. In a study by Yan et al. [50], a PDP test conducted on an AZ31 alloy in Hank’s solution reported an I_corr_ value of 2.74 × 10^−4^ A/cm^2^ (Table 3). However, the three Mg–MCA alloys showed lower I_corr_ values in Hank’s solution (Table 2), indicating excellent corrosion resistance. Further, Bakhsheshirad et al. [51] conducted an experiment on the biocorrosion and mechanical properties of the quaternary Mg–Ca–Mn–Zn alloy in a Kokubo (c-SBF) solution. The electrochemical test results reported that the corrosion potential values shifted more to the negative side with an increase in the concentration of the Mn and Zn content. The corrosion current density of the Mg–2Ca–0.5Mn–2Zn alloy was 78.3 × 10^−6^ A/cm^2^, the Mg–2Ca–0.5Mn–4Zn alloy was 99.6 × 10^−6^ A/cm^2^, and the Mg–2Ca–0.5Mn–7Zn alloy was 1.74.1 × 10^−6^ A/cm^2^ (Table 3). In the present investigation, it was observed that the three Mg–MCA alloys displayed very low I_corr_ values, indicating improved corrosion resistance. The low corrosion rates of the Mg–MCA alloys can be attributed to the presence of alloying elements, such as Cu and Mn, in addition to Mg, Al, and Zn, and their specific composition. Previous reports have shown that the addition of Al, Zn, and Mn improves the corrosion resistance of Mg alloys [52]. Additionally, the current Mg60, Mg70, and Mg80 alloys in all four SBF solutions showed, comparatively, very low I_corr_ values (Table 2). Thus, the present alloys, namely the Mg60, Mg70, and Mg80 alloys, exhibited good corrosion resistance compared to the previously reported alloys.

Among all three Mg–MCA alloys, the Mg60 alloy exhibited very low I_corr_ values (Table 2), indicating excellent corrosion resistance. The presence of Al, Zn, Mn, and Cu in the composition with less Mg content in the Mg60 alloy contributed to the improvement of corrosion resistance and the formation of a stable protective oxide layer. This resulted in a decrease in the I_corr_ value and hydrogen evolution rate, causing low corrosion rates in the Mg60 alloy, while high corrosion rates were observed in the Mg80 alloy. Overall, the novel Mg-based multicomponent alloys demonstrated excellent corrosion resistance compared to the previously reported alloys.

### 3.2. Electrochemical Impedance Spectroscopy (EIS) Measurement

**Figure 6:**
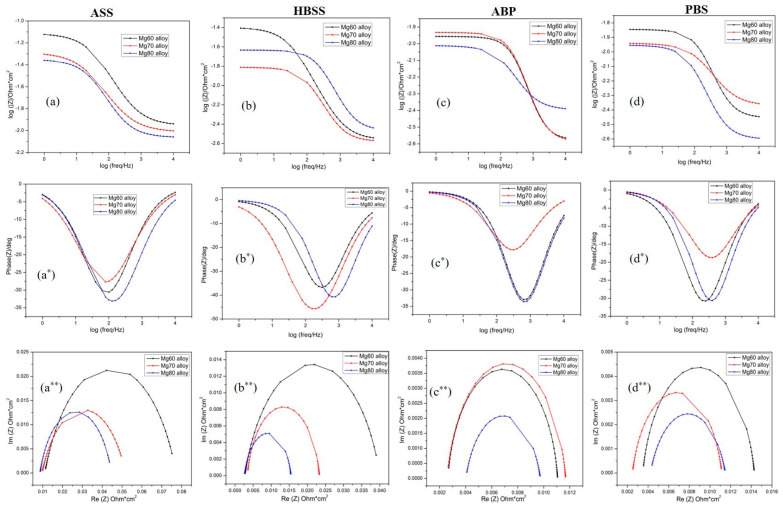
Bode plots [(**a**–**d**) represent log f vs. log Z; (a*–c*) represent log f vs. ϴ] and Nyquist plots [(a**–d**) represent the Z_real_ vs. Z_im_] for Mg60, Mg70, and Mg80 alloys in different body fluid solutions.

**Figure 7:**
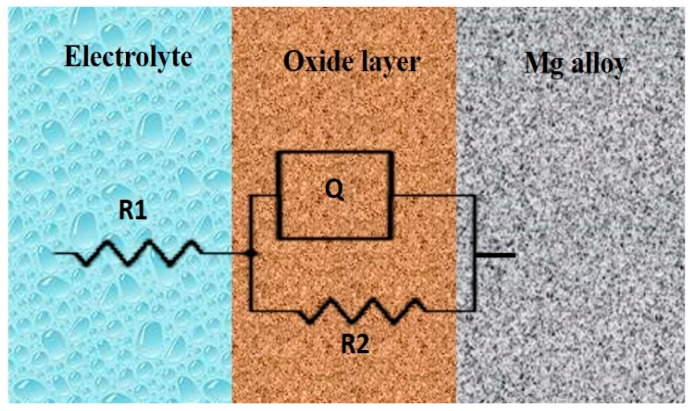
Randle’s equivalent circuit for Mg60, Mg70, and Mg80 alloys in simulated body fluids.

In addition to providing quantitative information on corrosion behavior, electrochemical impedance spectroscopy (EIS) measurements can offer insights into the corrosion process at the electrolyte/electrode interface and the changes in electrode properties. This information is crucial for comprehending the corrosion mechanism [53]. Examining the EIS behavior of Mg–MCA specimens, namely the Mg60 alloy, Mg70 alloy, and Mg80 alloy tested in various SBF solutions, the corresponding equivalent circuits for fitting the EIS data are proposed in Figure 6 and Figure 7, respectively. Figure 6 presents the EIS measurements in the Bode and Nyquist plots for the Mg–MCA samples under different SBF conditions. In Figure 6a, the Bode plots depict the impedance (Z) vs. the frequency (f), while Figure 6a* shows the phase angle (θ) vs. the frequency (f). Figure 6a** illustrates the Nyquist plot. The position of the absolute impedance |Z| in Figure 6a and the phase angle (θ) in Figure 6a* provide insights into the corrosion mechanisms. The Bode plot delineates the low-frequency region on the left-hand side and the high-frequency region on the right-hand side. Impedance analysis was conducted using an equivalent circuit.

Figure 6a–d represents the impedance vs. the frequency plot for the Mg60, Mg70, and Mg80 alloys in different body fluid solutions. The results reveal that the Mg60 alloy, while immersed in the ASS, HBSS, and PBS solutions, exhibits high impedance values in the low-frequency region, suggesting superior corrosion resistance compared to the other two alloys. As the Mg content increases from Mg60 to Mg70, there is a decrease in the absolute impedance values at the low-frequency region, indicating reduced passivation behavior. Whereas in the case of the ABP solution, compared to the Mg60 alloy, a high absolute impedance value |Z| observed for the Mg70 alloy, showing good passivation behavior, which corelates well with the polarization (Figure 1c) and SEM results in Figure 4. A further increase in the Mg content from the Mg70 to the Mg80 alloy, as seen in Figure 6a–d, shows that the absolute impedance values have moved to the more negative side, exhibiting significantly lower impedance values in the low-frequency region in all four SBF solutions, suggesting that high corrosion rates exist.

To provide further insight into the corrosion process, Figure 6a*,b*,d* displaces the Bode phase angle plot for the Mg–MCA alloys (Mg60, Mg70, and Mg80 alloys) under various SBF conditions. Generally, a high phase angle value in the low-frequency region and a smaller radius of the curvature signify a high corrosion rate [54]. Examining the plots, it is evident that the Mg60 alloy exhibits a high phase angle with a large radius of curvature in the ASS, HBSS, and PBS solutions in the high-frequency region, indicating superior corrosion resistance and good passivation behavior. This suggests that the alloy remains stable in these three solutions (ASS, HBSS, and PBS), followed by the Mg70 alloy. In the case of the Mg80 alloy, across all four SBF solutions, a small radius of curvature with a low phase angle is observed in the high-frequency region, indicating elevated corrosion rates. Furthermore, in the low-frequency region, the phase angle decreases to a lower value due to the degradation of the passive film [55,56]. Similar to the Bode absolute impedance plots, the phase angle plots for the Mg70 alloy in the ABP solution, as shown in Figure 6c*, show results comparable to the Mg60 alloy, signifying superior corrosion resistance in the ABP solution. No second-phase maximum is observed in any case, indicating the presence of a stable protective layer on the surface of the three Mg–MCA alloys. The Bode plot analysis highlights that the Mg60 alloy is more stable, whereas the Mg80 alloy is more soluble in all four SBF solutions. Notably, all three alloys exhibit excellent corrosion resistance in the ASS solution, consistent with the linear polarization results in Figure 1.

Figure 6a**–d** shows the Nyquist plot of the Mg–Al alloy in different SBF solutions. Evidently, the Mg60 alloy in the ASS solution exhibits high imaginary values with a large radius of curvature, surpassing the other two alloys, Mg70 and Mg80. This substantial radius of curvature and elevated imaginary values for the Mg60 alloy signify high corrosion resistance, indicating the formation of a strong passive film, subsequently followed by the Mg70 alloy. The Mg80 alloy, as shown in Figure 6c**, displays a small radius of curvature with lower imaginary values in the low-frequency region across all four SBF solutions, representing a high corrosion rate. The escalation in the corrosion rate is mainly attributed to the presence of alloying elements and an increased chlorine ion concentration. This implies that the alloy absorbs chloride ions on the surface, leading to increased oxidation by consuming metal ions. Consequently, surface degradation, pit initiation, and crack formation on the alloy surface are evident, as observed in the SEM images of the Mg80 alloy (Figure 3, Figure 4 and Figure 5). In the case of the ABP solution, the Mg70 alloy exhibits high corrosion resistance compared to the Mg60 alloy, owing to the elevated concentration of Cl^−^, along with PO_4_^3−^ and SO_4_^2−^ ions. The impedance results for the Mg60, Mg70, and Mg80 alloys in the SBF solution, as shown in Figure 6, align well with the polarization curves in Figure 1 and the SEM results in Figure 3, Figure 4 and Figure 5.

Figure 7 represents the equivalent circuit for the Nyquist plots of the Mg–MCA (Mg60, Mg70, and Mg80 alloys) samples tested in the SBF solutions with a circuit description code of R1 + Q/R2. The circuit configuration was obtained through the EC-Lab software. The electrochemical parameters derived from the EIS fitting analysis for the SBF solutions are outlined in Table 4. The equivalent circuit symbolizes a reaction involving the metal/oxide layer/electrolyte. The electrochemical parameters obtained include the solution resistor (R1), representing the interface between the reference electrode and the counter electrode. The charge transfer resistor (R2) signifies the metal substrate/solution interface. The constant phase element (Q) acts as an imperfect capacitor, due to the inhomogeneous microstructure, roughness, and composition of the alloy surface layer. The constant phase element exponential (n) ranges from 0 to 1, with a value of 1 indicating that the CPE is acting as a capacitor and a value of 0 indicating that the CPE is acting as an ideal resister.

From Table 4, it is evident that in the ASS solution at pH 6.2, the charge transfer resistor (R2) for the Mg60 alloy is notably higher. This value surpasses those for the Mg70 and Mg80 alloys, indicating a higher resistance to corrosion and the formation of a robust passivation layer. As the Mg content increases from Mg60 to Mg70, there is a discernible decrease in the R2 values, signifying lower resistance to passivation and an increase in the corrosion rate. In the case of the Mg80 alloy, with a further increase in the Mg content, very low R2 values are observed. This implies that the alloy fails to form a protective layer on the surface, resulting in material degradation and heightened corrosion rates. The order of the corrosion rate is as follows: Mg80 > Mg70 > Mg60 alloys.

As the pH value increases from 6.2 to 7.4 in the HBSS solution, the Mg60 alloy exhibits significantly lower R2 values (Table 4) compared to the ASS solution, followed by the Mg70 and Mg80 alloys. This trend is similarly observed in the PBS solution at a pH level of 7.4. The observed behavior can be attributed to an increase in Cl^−^, SO_4_^2−^, and PO_4_^3−^ ions, along with a decrease in the pH value (7.4 to 6.2) from the PBS solution to the ASS solution. In contrast, in the case of the ABP solution (Table 4), the Mg70 alloy demonstrates high and more stable corrosion resistance compared to the Mg60 alloy, followed by the Mg80 alloy.

From Table 4, it is observed that the CPE exponential value ‘n’ ranges between 0.5 and 1 for all four SBF solutions, indicating Warburg impedance with diffusion properties and the formation of a passive layer. With a further increase in the Cl^−^ ion concentration from pH 6.2 to pH 7.4 in the SBF solutions, there is a decrease in the charge transfer resistor (R2) values. This signifies that the corrosion resistance of the alloy decreases with an increase in the Cl^−^ ion concentration, resulting in less protection from corrosion. Additionally, more pores/defects are noticeable on the alloy surface (Figure 3, Figure 4 and Figure 5), indicating higher corrosion rates. Notably, Table 4 shows that the CPE exponent value ‘n’ for the ABP, PBS, and HBSS solutions is close to 1, indicating ideal capacitor behavior. Interestingly, the ASS solution shows a lower n value (0.7) compared to the other three solutions. The measurements of the R2 and Q1 values align well with the polarization method (Figure 1) and SEM results (Figure 3, Figure 4 and Figure 5). This suggests that the ASS solution provides better corrosion resistance to the alloy compared to the ABP, HBSS, and PBS solutions.

### 3.3. Energy-Dispersion X-ray Spectroscopy

To complement the polarization results (Figure 1), SEM analysis (Figure 3, Figure 4 and Figure 5), and EIS (Figure 6) analysis, the simulated body fluid samples of the Mg–MCA alloys (Mg60, Mg70, and Mg80 alloys) were subjected to energy-dispersion X-ray spectroscopy (EDS). The chemical composition, expressed in atomic percentage, is presented in Table 5. In the case of samples tested in the ASS solution, Table 5 reveals the presence of O, Mg, Al, Zn, Cu, Mn, and P. The composition in Table 5 reveals that in the Mg60 alloy, oxygen (at.%) is found to be 14.04 at.% and Mg is 60.49 at.%, along with the other alloying elements. Further, in relation to the increase in the percentage of Mg content from the Mg60 alloy to the Mg70 alloy, the O content is 17.10% and Mg is 59.64%. Notably, in the Mg80 alloy, there is a significant reduction in the Mg, Al, Zn Mn, and Cu alloy content, with a drastic increment in the O at% (17.10 to 36.65%) content, as shown in Table 5. Comparing all the three alloys in the ASS solution at pH 6.4, a significantly lower amount of O at.% and a higher amount of Mg %.at is observed in the Mg60 alloy. This means that Mg60 alloy displays good corrosion resistance and high corrosion rates are observed in the Mg80 alloy.

In aqueous solution, when the sample is immersed, magnesium is oxidized and forms Mg^+^, as shown in Equation (1). The formation of Mg^+^ occurs due to the dissolution of Mg and Mg^+^. Based on Equation (1), anodic dissolution is evident in this process, primarily associated with magnesium dissolution. The cathodic reaction is depicted in Equation (2), which involves the generation of OH^−^ concurrently with the liberation of H_2_ from the surface (cathode).

(1) $$Anodic\;Reaction:Mg\to {Mg}^{2+}+{2e}^{-}\;$$

(2) $$Cathodic\;Reaction:{2H}_{2}O+{2e}^{-}\to {2OH}^{-}+H_{2}$$

The metal Mg^+^ produced reacts with water, releasing hydrogen bubbles from the alloy. Mg ions and the liberated OH^−^ combine to form a protective MgO/Mg(OH)_2_ layer on the alloy’s surface, which is illustrated in Equations (3) and (4). This forms a protective layer, which is highly stable and results in lower corrosion rates.

(3) $${Mg}^{2+}+{2H}_{2}O\to MgO+{2H}^{+}$$

(4) $$or\;{Mg}^{2+}+2{OH}^{-}\to Mg{\left(OH\right)}_{2}+{2e}^{-}$$

**Figure 8:**
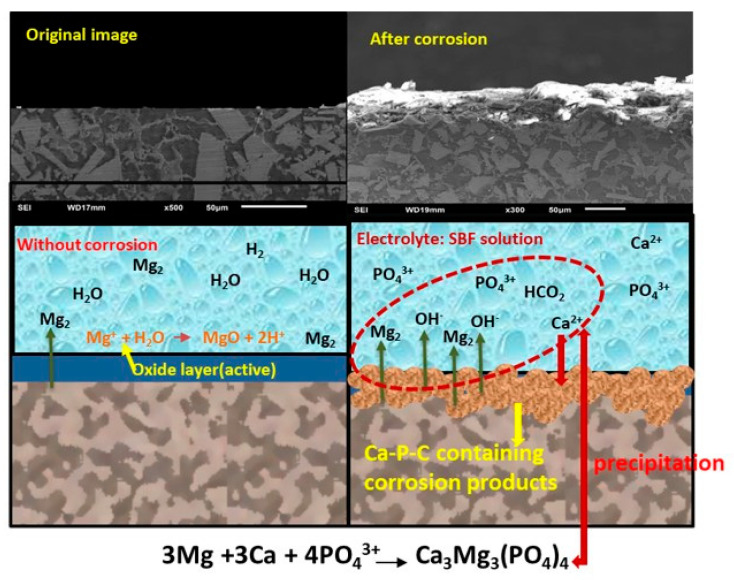
Schematic representative model showing the corrosion mechanism of the magnesium alloy in SBF solution.

The formed magnesium hydroxide layer (MgO/Mg(OH)_2_) is a natural occurrence and serves as a protective later for underlying metal. This layer can prevent future corrosion and degradation of magnesium. The formation of the protective layer and the corrosion process can be observed from the schematic illustration in Figure 8. However, in certain corrosive environmental conditions, alloys can undergo corrosion and form other compounds. During the analysis of the three Mg–MCA samples immersed in the ASS solution (Table 1), in the Mg60 alloy, Mg dissolves, releasing hydrogen bubbles from the alloy. The Mg ions and released OH^−^ ions combine to form Mg(OH)_2_, as shown in Equation (4). This formed protective surface layer of Mg(OH)_2_ is highly stable and provides excellent protection to the alloy. Additionally, the low concentration of Cl^−^ ions and the absence of phosphorus (P) and potassium (K) prevents the dissolution of a passive layer, resulting in excellent corrosion resistance. Whereas for the Mg80 alloy in the ASS solution, it can be observed that there is a drastic increment in the O at.% (17.10 to 36.65%) content, along with a reduction in the Mg and other alloying elements (Table 5). With a high O content and Cl^−^ ion concentration in the ASS solution, Mg(OH)_2_ breaks downs as the highly concentrated Cl^−^ ions react with Mg(OH)_2_. Cl^−^ ions replace OH^−^ ions, resulting in the conversion of Mg(OH)_2_ to MgCl_2_, as shown in Equation (5). This breakdown of the protective layer leads to the dissolution of α-Mg, causing the alloy surface to be covered with irregular pits and deeper cracks (SEM: Figure 5). This is mainly attributed to the loss of water content from the surface of the alloy, leading to surface shrinkage.

(5) $${Mg(OH)}_{2}+{2Cl}_{2}^{-}\to {MgCl}_{2}+{2H}_{2}O$$

In the case of the Mg60 and Mg70 alloys, no surface damage was observed on the alloy surface (SEM: Figure 3), indicating that the protective Mg(OH)_2_ layers are completely stable and resistant to further corrosion. This is mainly due to the formation of Mg_32_(AlZn)_49_ and Mg_17_Al_12_ phases_._ The addition of Mn and Cu to Mg alloys play a crucial role in reducing corrosion rates.

Further, the EDS and chemical composition analyses were conducted on the Mg60, Mg70, and Mg80 alloy samples tested in the HBSS solution. As the pH levels increased from pH 6.4 to 7.4, there was a significant rise in the O %.at content, coupled with a notable reduction in the Mg, Al, Zn, Cu, Mn, and P content in all the alloys. This phenomenon is primarily attributed to the presence of a high level of Cl^−^, SO_4_^2−^, and PO_4_^3−^ ions in the solution. These ions contribute to the breakdown of the passive layer (Mg(OH)_2_) and cause the increased dissolution of α-Mg, forming a MgCl_2_ layer. The released OH^−^ ions result in an increase in the pH value of the solution, leading to the formation of corrosive products with fine cracks on the alloy surface, indicating high corrosion rates in the HBSS solution, as observed in the SEM images in Figure 3, Figure 4 and Figure 5. The corresponding chemical reactions are shown in Equations (1)–(5). It has been reported that high pH values accelerate the precipitation of calcium (Ca^2+^) and phosphate (PO_4_^3+^) in HBSS solutions. Consequently, magnesium dissolution increases with an elevated pH value, leading to the precipitation of Ca_3_Mg_3_(PO_4_)_4_ (Figure 8 and Equation (6)) [57]. The corrosion rates of the Mg60, Mg70, and Mg80 alloys are higher in the HBSS solution compared to the ASS solution.

(6) $$3Mg+3Ca+{4PO}_{4}^{3-}\to {Ca}_{3}{Mg}_{3}{(PO}_{4}{)}_{4}$$

In the case of the Mg alloys in the PBS solution, the EDS results reveal a significant reduction in Mg, Al, Zn, Mn, and Cu, accompanied by an increase in oxygen (O), compared to the ASS and HBSS solutions. With an increase in the Mg content from the Mg60 alloy to the Mg80 alloy, the O %.at increased from 53.32% to 61.27% (Table 5). When the sample is immersed in the PBS solution, Mg^2+^ ions react with Ca and PO_4_^3−^ particles to form a white compound, namely Ca_3_Mg_3_(PO_4_)_4_, as shown in Equation (6). Due to the high concentration of P and K particles in the PBS solution, it causes the dissolution of the alloy with the evolution of hydrogen, leading to an increase in the concentration of OH^−^ ions. The OH^−^ ions formed at the substrate interface (Equation (5)) reduce H_2_PO_4_^−^ and HPO_4_^2−^ (in SBF) into PO_4_^3−^ ions, as shown in Equations (7) and (8).

(7) $$H_{2}{PO}_{4}^{-}+{2OH}^{-}\to {PO}_{4}^{3-}+{2H}_{2}O$$

(8) $$and\;{2HPO}_{4}^{2-}+{2OH}^{-}\to {2PO}_{4}^{3-}+{2H}_{2}O$$

(9) $$6MgO+{2PO}_{4}^{3-}+O_{2}\to {Mg}_{3}{(PO}_{4}{)}_{2}$$

The PO_4_^3−^ ions formed adhere to the pores in the primary corrosion product Mg(OH)_2_/MgO film, resulting in the formation of the white compound (Mg_3_(PO_4_)_2_) [57], as illustrated in Equation (9). This secondary corrosive product (Mg_3_(PO_4_)_2_) covers the reacted surface, diminishes the electrochemical activity of the substrate, and decelerates the corrosion rate, contributing to a slight improvement in the corrosion resistance of the alloy. Conversely, a substantial amount of Cl^−^ ions is adsorbed into the weak portions of the film layer, forming soluble magnesium chloride (MgCl_2_), as indicated in Equation (5). Consequently, numerous surface degradations with cracks are observed on the alloy surface in the PBS media (SEM: Figure 3, Figure 4 and Figure 5). Based on the increase in the oxygen concentration, higher corrosion rates, along with more degradation, are observed in the Mg80 alloy in all the solutions, while the Mg60 alloy exhibits comparatively less degradation.

The EDS results obtained for the ASS, HBSS, and PBS solutions are in line with the polarization (Figure 1 and Figure 2) and SEM results (Figure 3, Figure 4 and Figure 5). The EDS analysis of the samples tested in the ABP solution revealed that, compared to the Mg60 alloy, the Mg70 alloy exhibited lower oxygen content at 30.61% and higher magnesium content at 48.44 at.%. This suggests that the Mg70 alloy demonstrates superior corrosion resistance in the ABP solution, indicating lower reactivity compared to the Mg60 alloy. Conversely, an increase in the magnesium content leads to heightened corrosion behavior in the Mg80 alloy.

In a study akin to the current analysis, Heakal et al. [57] investigated the degradation behavior of the AZ80E magnesium alloy in phosphate buffer saline solutions. EDS mapping clearly indicated that initially, the alloy exhibited low corrosion rates due to the precipitation of PO_4_^3−^ ions into the primary corrosion products, namely Mg(OH)_2_ and (Mg_3_(PO_4_)_2_, leading to self-healing and enhanced corrosion resistance. However, after a certain exposed period, aggressive Cl^−^ ions weakened the film, leading to an increase in the corrosion rate. Similarly, Weiwei He et al. [58] delved into the biocorrosion behavior of the extruded Mg–Zn–Mn alloy in Hank’s solution. The EDS mapping distinctly showed a high oxygen content in correlation with an increase in the Cl^−^ ion concentration, providing evidence of the corrosion mechanism in the Mg–Zn–Mn alloy.

Among the three Mg–MCA samples immersed in the four SBF solutions, the Mg60 alloy showed excellent corrosion resistance in the ASS, HBSS, and PBS solutions, except in the ABP solution. In addition to this, the Mg60 alloy in the ASS solution also showed outstanding corrosion resistance with good passivation behavior. This exceptional performance can be attributed to the presence of alloying elements, with their specific chemical composition, and a lower level of Mg content. Along with the chemical composition, another reason to prevent dissolution of the protective layer is the formation of secondary phases, such as Mg_32_(AlZn)_49_ and Mg_17_Al_12_ phases. These phases in the alloy help to reduce the corrosion rate. Additionally, the binary addition of Cu and Mn to the Mg–Al alloy in the Mg–Al–Zn system, results in a stable protective film on the alloy surface, leading to excellent corrosion resistance in the ASS solution.

Furthermore, a crucial factor contributing to the improved corrosion resistance of the Mg60 alloy is the presence of manganese (Mn) and copper (Cu), alongside Mg, Al, and Zn. Manganese, within the Mg matrix, exerts a positive influence on the corrosion behavior. Manganese (Mn) undergoes a reaction with water to form manganese oxide, as depicted in Equation (10). It is anticipated that the absorption of oxidized manganese by magnesium hydroxide through the substitution of magnesium cations, hinders the penetration of chloride anions into the magnesium hydroxide, thereby enhancing the corrosion resistance of the alloy.

(10) $$Mn+{4OH}^{-}\to {2H}_{2}O+MnO_{2}$$

Simultaneously, when the Mg alloys are immersed in the aqueous solution, copper ions within the Mg alloy (Cu) react with water molecules, initially converting to copper hydroxide (Cu(HO)_2_) (Equation (11)), which serves as a protective agent against corrosion. This subsequently leads to the formation of copper oxide (CuO) (Equation (12)).

(11) $${Cu}^{2+}+{2OH}^{-}\to {Cu\left(OH\right)}_{2}$$

(12) $${Cu\left(OH\right)}_{2}\to CuO+{2H}_{2}O$$

(13) $$2Cu+{2Cl}^{-}+{3OH}^{-}\to {Cu}_{2}Cl{\left(OH\right)}_{3}+{4e}^{-}$$

However, when the copper is exposed to corrosive environments that contain Cl^−^, SO_4_^2−^, and PO_4_^3−^ ions, it can undergo corrosion and form copper hydroxy chloride (Cu_2_Cl(OH)_3_) (Equation (13)). Based on the EDS analysis, when the Mg alloy sample was exposed to the ASS solution at pH 6.2 (Table 5), even though the Cu content showed higher values, the best corrosion results were observed among the three alloys, due to the lower Cl^−^ ion concentration in the absence of SO_4_^2−^ and PO_4_^3−^ ions (Table 1).

Nevertheless, with an increase in the pH level from 6.4 to 7.4, in the ABP, PBS, and HBSS solutions (Table 1), more corrosive products and cracks were observed on the sample surface, which can be seen in the SEM images (Figure 3, Figure 4 and Figure 5).This could be due to the high halide ion concentration in the electrolyte solution (Table 1). When the alloy reacts with the HBSS, ABP, and PBS solutions, Al_6_Mn, Al_2_CuMg, and Al_2_Cu phases were detected [59]. These phases are more active in a halide environment and particles are expected to dissolve more rapidly. This leads to the development of corrosion defects, along with cracks on the alloy surface, indicating high corrosion rates. The results reveal good correlation between the linear polarization (Figure 1 and Figure 2), SEM (Figure 3, Figure 4 and Figure 5), and EDS (Table 5) results.

## 4. Cytotoxicity Behavior of Mg–MCA Alloys

Generally, after prosthesis implantation, tumor reappearance and spread pose significant changes in the medical field. Therefore, it is imperative to develop implant materials with antitumor properties. Magnesium (Mg) alloys exhibit excellent biocompatibility, mechanical properties, and biodegradability in implant applications. However, there have been limited reports on whether they possess anticancer properties. In addition to studies on different types of simulated body fluids, interesting behavior of Mg alloys has been observed in cell cultured analysis. Recent studies have focused on the development of Zn and Mn-based Mg alloys due to their biocompatibility and osteogenic activities, and these alloys have demonstrated good antitumor properties [60]. Consequently, in vitro cytotoxic tests were conducted on Mg-based multicomponent alloys (Mg60, Mg70, and Mg80 alloys) to investigate the antitumor properties using MDA-MB-231 cells.

**Figure 9:**
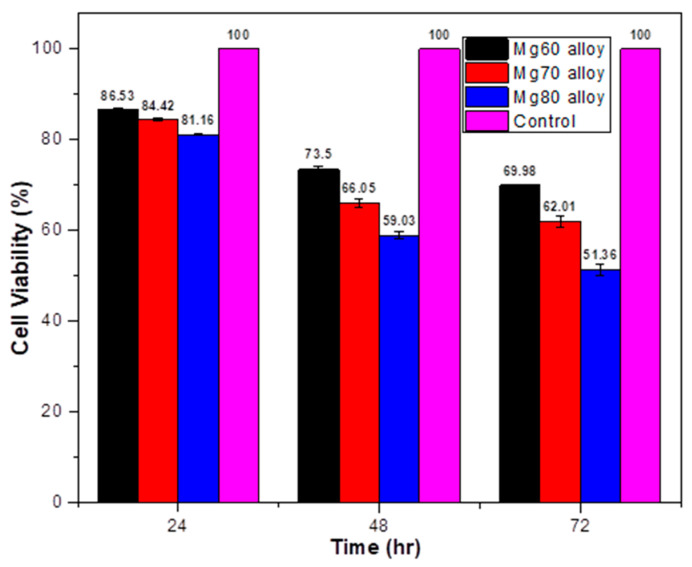
The cytotoxicity of MDA-MB-231 cells cultured in Mg60, Mg70, and Mg80 alloys.

The proliferation ability of human breast tumor cell (MDA-MB-231) lines cultured in Mg60, Mg70, and Mg80 alloys is depicted in Figure 9 and Table 6. In comparison to the control, all the Mg alloys demonstrated a reduction in the cell number (Table 6). As we can see from Figure 9 and Table 6, at 24 h of incubation, the Mg60 alloy exhibited high cell viability at 86.24%. With an increase in the Mg content from Mg60 to Mg70, there was a decrease in the cell viability from 86.24% to 84.4%, followed by the Mg80 alloy. Compared to the Mg60 alloy, less cell viability was observed in regard to the Mg80 alloy (81.21%) after 24 h of incubation. Over the incubation period from 24 h to 48 h (day 2), the cell viability decreased with the increment in the Mg content in regard to the three alloys. Once again, the Mg80 alloy exhibited the lowest cell viability at 59.29%. By day 3 (72 h), all three Mg alloys showed significantly lower cell proliferation. Throughout the entire incubation period, the Mg60 alloy demonstrated high cell viability, while the Mg80 alloy exhibited low cell viability, i.e., 51.64%.

**Figure 10:**
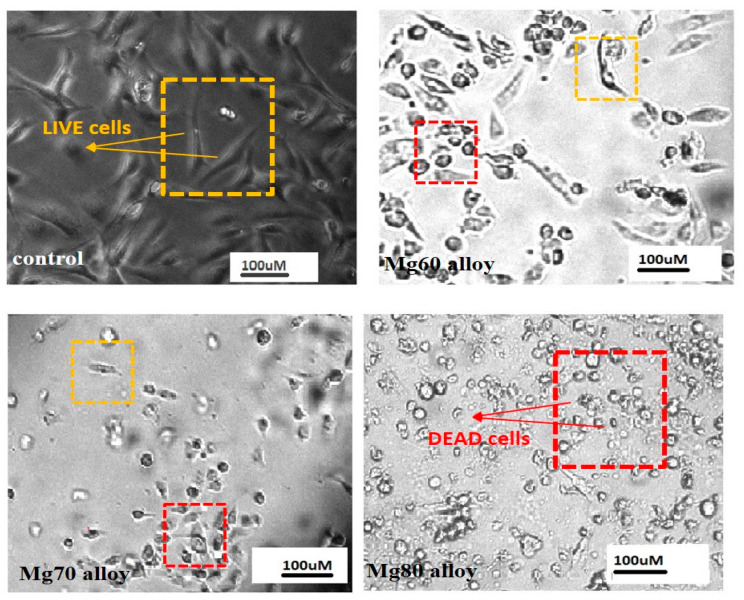
Microscopic view of cell morphologies of MDA-MB-231 cells cultured in Mg60, Mg70, and Mg80 alloys on day 3 (72 h) and cell density (live cells—yellow square box, dead cells—red square box).

The cell morphologies were observed using inverted phased contrast microscopy. Live/dead cells of MDA-MB-231 were identified based on cellular morphology with spindle shaped cells indicating live whereas spherical or round shaped cells indicating dead. These images were obtained from cells cultured in Mg60, Mg70, and Mg80 alloys on day 3 (72 h) (Figure 10). For quantitative analysis, in comparison to the control group, all the Mg alloy groups demonstrated a reduction in the cell number, but most cells were alive (in yellow square box) in the Mg60 alloy extract. Cells cultured in Mg-containing alloys (Mg70 and Mg80 alloys) exhibited unhealthy shrinkage shapes (displayed in Figure 10). Additionally, more dead cells can be observed in regard to the Mg80 alloy (in red square box). Consistent with the MTT assay, a higher Mg content in the alloy showed better inhibition efficiency in terms of proliferation and vitality.

Based on the in vitro cytotoxicity results, it can be observed that Mg along with Zn and Mn containing alloys significantly inhibit the growth and viability of the MDA-MB-231 cells (Figure 9 and Figure 10). The observed cytotoxic effect can be correlated with the polarization results and SEM results. A relatively fast degradation of the material can enhance the release of elements; hence, these elements support anticancer activity [60].

In accordance with the above observation, the biocorrosion process releases Zn ions into the cultured medium, impeding the proliferation of tumor cells by altering the cell cycle and inducing cell apoptosis through the activation of the mitochondrial membrane potential and caspase cell cycle [60]. Furthermore, Mn exhibits no toxic effect and plays a crucial role in activating various enzymes, such as hydrolases, kinases, transferases, decarboxylases, and mitochondrial respiration [61,62]. In conjunction with Mn and Zn, Mg also plays a significant role in suppressing tumor cells. According to reports [63], the degradation of Mg leads to an increase in the pH level, thereby suppressing/damaging bacterial growth due to the osmotic shock they experience [60]. Chen et al. [64] reported that anodic oxidation with heat-treated Mg alloys suppressed breast cancer cells in both in vitro and in vivo studies. Hakimi O et al. [65] conducted an experiment using the biodegradable EW62 alloy in a medical implant application to resist tumor cell growth. The results indicated that the EW62 alloy displayed the best antitumor properties. Natalia Anisimova et al. [61] studied the cytotoxicity of the biodegradable magnesium alloy WE43 in regard to tumor cells, such as LNCaP and MDA-MB-231 cells. The alloys were developed by using severe plastic deformation using the ECAP technique. The results demonstrated that alloy the WE43 alloy can be considered as a promising material for orthopedic implant applications in clinical oncology. Thus, the data presented above demonstrate that the Mg80 alloy does exhibit an inhibitory effect on MDA-MB-321 tumor cells. This is due to the presence of Zn and Mn ions in the biocorrosion process, which contributes to inhibiting tumor cell growth and inducing cell apoptosis, supporting the observed antitumor activity of the Mg80 alloy.

The natural question arising here is whether the observed cytotoxicity effect is attributable to the presence of the alloying elements or the base material, magnesium. Currently there is insufficient evidence to conclusively address this issue. According to previous reports, the presence of Zn and Mn ions in the cultured medium during the biocorrosion process delays the proliferation of tumor cells and induces cell apoptosis [48]. Further investigations are necessary to determine whether Mg alone is the primary factor or whether it merely supports the alloying elements in the Mg80 alloy. Regardless of the root cause of the effect, the current results have demonstrated that the Mg80 alloy holds promise as a material for implant applications in clinical oncology. It could fulfill a dual role as a mechanically stable, yet bioresorbable scaffold, with localized antitumor activity.

## 5. Conclusions

In this research, an in-depth examination of the corrosion behavior and microstructure of as-cast Mg–MCA alloys in diverse simulated body fluid solutions were studied. The alloys confirmed commendable corrosion resistance across all the SBF solutions, surpassing the performance of previously reported alloys. Furthermore, we delved into the antitumor properties by assessing the cytotoxic activity against DA-MB-231 cancer cells.

From the findings of the current study, several conclusions can be drawn, as follows:

1. The electrochemical corrosion behavior of the Mg–MCA alloys were tested in various simulated body fluids, and the corrosion resistance order for the Mg–MCA alloys is as follows: ASS > HBSS > ABP > PBS;
2. Among all the three alloys, the Mg60 alloy exhibited low corrosion rates (~× 10^−5^ mm/year), with negligible surface defects observed on the alloy surface in all solutions. This can be attributed to the formation of Mg_32_(AlZn)_49_ and Mg–Zn–Cu phases and a lower Mg content, with a high atomic percentage of alloying elements. This stable nature contributes to excellent corrosion resistance;
3. The Mg80 alloy displayed high corrosion rates (range from 7.97 × 10^−5^ to 0.74 mm/year) and severe surface degradation, with deeper cracks observed on the alloy surface when immersed in all four solutions, leading to higher metal dissolution. This can be attributed to the high Mg content and the formation of intermetallic phases, such as Al_6_Mn, Al_2_CuMg, and Al_2_Cu phases;
4. It is interesting to note that in the ABP solution, the Mg70 alloy displayed a high corrosion resistance value (5.84 × 10^−5^ mm/year) compared to the Mg60 alloy (1.68 × 10^−4^ mm/year);
5. According to the EDS analysis, which further validates the polarization, SEM, and impedance data parameters, all three Mg–MCA alloys in the ASS solution exhibited superior corrosion resistance. This strongly indicates that the current alloys are exceptionally well-suited for dental applications;
6. In vitro studies revealed that the novel Mg–MCA alloys demonstrate cytotoxicity activity against tumor cells;
7. Analysis of the live/dead morphologies in the occurrence of MDA-MB-231 cells showed an increased presence of dead cells in regard to the Mg80 alloy, with majority of live cells observed in regard to the Mg60 alloy;
8. In conclusion, the results indicate that, concerning proliferation and vitality, the Mg80 alloy can be deemed a promising material with potential antitumor activity for implants.

## Figures and Tables

**Table 1:** Chemical composition of different simulated body fluids.

Contents (g/L)	ABP (pH 7.4)	HBSS (pH 7.4)	PBS (pH 7.4)	ASS (pH 6.2)
NaCl	8.036	8.0	8.0	1.5
NaHCO_3_	0.352	0.35	-	1.5
NaH_2_PO_4_	-	-	1.15	0.5
KCl	0.225	0.4	0.2	-
KSCN	-	-	-	0.5
KH_2_PO_4_	-	0.06	0.2	-
Lactic Acid	-	-	-	0.9
Na_2_HPO_4_·3H_2_O	0.238	-	-	-
MgCl_2_·6H_2_O	0.311	-	-	-
CaCl_2_	0.293	-	-	-
Na_2_SO_4_	0.0072	-	-	-
CaCl_2_·2H_2_O	-	0.19	-	-
MgSO_4_·7H_2_O	-	0.2	-	-
Na_2_HPO_4_·7H_2_O	-	0.09	-	-
Glucose	-	1.0	-	-

**Table 2:** Corrosion current density (I_corr_) and potential (E_corr_) of Mg–MCA in SBF solutions.

Type of Solution	Material	I_corr_ (A/cm^2^)	E_corr_ (V vs. SCE)
ASS	Mg60 alloy	3.48 × 10^−10^	−1.19
	Mg70 alloy	2.03 × 10^−9^	−1.22
	Mg80 alloy	4.68 × 10^−9^	−1.29
PBS	Mg60 alloy	4.30 × 10^−9^	−1.15
	Mg70 alloy	1.97 × 10^−8^	−1.20
	Mg80 alloy	4.40 × 10^−5^	−1.48
ABP	Mg60 alloy	1.15 × 10^−8^	−1.23
	Mg70 alloy	3.57 × 10^−9^	−1.21
	Mg80 alloy	9.99 × 10^−6^	−1.39
HBSS	Mg60 alloy	8.55 × 10^−10^	−1.25
	Mg70 alloy	3.52 × 10^−9^	−1.23
	Mg80 alloy	1.81 × 10^−8^	−1.29

**Table 3:** The current density (I_corr_, A/cm^2^) of previously reported Mg alloys in different SBF solutions [49,50,51].

Composition	Solution	pH Level	I_corr_ (A/cm^2^)
Mg–Mn–Zn alloy	PBS	7.4	7.917 × 10^−5^
AZ31 alloy	HBSS	7.4	2.74 × 10^−4^
Mg–2Ca–0.5Mn–2Zn alloy	Kokubo (c–SBF)	7.6	78.3 × 10^−6^
Mg–2Ca–0.5Mn–4Zn alloy		7.6	99.6 × 10^−6^
Mg–2Ca–0.5Mn–7Zn alloy		7.6	174.1

**Table 4:** Electrochemical parameters of Mg60, Mg70, and Mg80 alloys in simulated body fluids from the impedance fitting diagram.

Solution	Alloy	R1 (Ω)	R2 (Ω)	Q1 (F)	n
ASS	Mg60	0.01	0.06	0.27	0.7
	Mg70	9.66 × 10^−3^	0.04	0.67	0.7
	Mg80	8.61 × 10^−3^	0.035	0.46	0.7
HBSS	Mg60	3.42 × 10^−3^	0.019	0.078	0.8
	Mg70	3.49 × 10^−3^	0.01	0.22	0.8
	Mg80	4.23 × 10^−3^	7.21 × 10^−3^	0.63	0.7
ABP	Mg60	2.63 × 10^−3^	8.42 × 10^−3^	0.124	0.9
	Mg70	2.57 × 10^−3^	9.10 × 10^−3^	0.13	0.8
	Mg80	4.01 × 10^−3^	5.72 × 10^−3^	0.65	0.7
PBS	Mg60	2.71 × 10^−3^	0.03	0.38	0.7
	Mg70	2.62 × 10^−3^	0.01	0.28	0.8
	Mg80	2.49 × 10^−3^	8.65 × 10^−3^	0.55	0.8

**Table 5:** EDS chemical mapping of Mg60, Mg70, and Mg80 alloys in simulated body fluids.

Solution	Alloy	O (%.at)	Mg (%.at)	Al (%.at)	Zn (%.at)	Cu (%.at)	Mn (%.at)	P (%.at)	Cl (%.at)
ASS	Mg60	14.04	60.49	12.71	5.35	4.12	2.84	0.46	-
	Mg70	17.10	59.64	13.07	3.46	6.18	0.55	-	-
	Mg80	36.65	52.34	1.89	3.79	4.11	0.31	0.90	-
HBSS	Mg60	40.41	38.64	9.58	3.42	6.22	1.13	0.59	-
	Mg70	44.73	43.05	5.64	3.00	2.32	0.58	0.68	-
	Mg80	71.56	28.33	0.11	0.00	0.00	0.00	0.00	-
ABP	Mg60	60.71	29.78	3.79	1.45	2.41	0.94	0.94	-
	Mg70	30.61	48.44	8.75	4.05	2.76	1.09	4.29	-
	Mg80	70.80	28.48	0.19	0.25	0.22	0.04	0.03	-
PBS	Mg60	53.32	36.46	4.01	2.36	1.50	0.55	2.12	-
	Mg70	61.27	29.06	3.97	1.28	2.89	0.97	-	0.55
	Mg80	70.53	28.80	0.19	0.12	0.08	0.04	0.25	-

**Table 6:** Cell viability of MDA-MB-231 cells cultured in Mg60, Mg70, and Mg80 alloys.

Time	Alloy	Cell Viability (%)	Standard Deviation % CV
24 h	Mg60	86.24	0.37
	Mg70	84.4	0.37
	Mg80	81.21	0.12
48 h	Mg60	72.91	0.69
	Mg70	65.72	0.80
	Mg80	59.29	0.77
72 h	Mg60	70.14	0.17
	Mg70	61.16	1.36
	Mg80	51.64	1.16

## Data Availability

Data are available on request from the authors.
